# Greener care, smarter spending: economic opportunities in sustainable nephrology

**DOI:** 10.1093/ckj/sfag259

**Published:** 2026-08-07

**Authors:** Ivo Laranjinha, Anna Julie Peired, Susi Knoeller, Sonja Gracin, Ana Carina Ferreira, Maryvonne Hourmant, Gulay Demirtas

**Affiliations:** Nephrology Department, Unidade Local de Saúde de Lisboa Ocidental, Lisbon, Portugal; Nephrology and Dialysis Unit, Meyer Children’s Hospital IRCCS, Florence, Italy; Department of Nephrology and Transplantation, GesundheitNord, Klinikum Bremen Mitte, Bremen, Germany; Transplantation Centre, Sahlgrenska University Hospital, Gothenbourg, Sweden; Department of Nephrology, Hospital Curry Cabral – Unidade Local de Saúde de São José, Lisbon, Portugal; Nova Medical School, Lisbon, Portugal; Service de Néphrologie et Immunologie Clinique, University Hospital of Nantes, Nantes, France; Department of Nephrology, SBU Antalya Training and Research Hospital, Antalya, Türkiye

**Keywords:** chronic renal failure, chronic renal insufficiency, CKD, dialysis, hemodiafiltration

## Abstract

Sustainable nephrology should be recognized not only as an environmental priority but also as an economic and clinical imperative. Kidney care, particularly dialysis, is among the most resource-intensive areas of healthcare, generating substantial costs and environmental impacts through high consumption of water, energy, plastics, and pharmaceuticals. Increasing evidence suggests that strategies such as chronic kidney disease prevention, risk-based follow-up, conservative kidney management, incremental dialysis, lean dialysis practices, circular resource management, and telenephrology can simultaneously reduce resource use, greenhouse gas emissions, and healthcare expenditure while maintaining or improving patient outcomes. Real-world experiences further demonstrate that sustainability initiatives can deliver substantial carbon reductions alongside significant financial savings. However, achieving these benefits requires systematic measurement of environmental performance, life-cycle assessment of technologies, and reimbursement models that reward value, prevention, and resource efficiency rather than healthcare volume. Sustainable nephrology therefore represents an opportunity to align environmental stewardship with economic sustainability and high-quality patient care. Integrating sustainability into kidney care is not simply an ecological responsibility but a practical strategy to improve the long-term resilience, efficiency, and value of health systems.

Sustainable nephrology should no longer be viewed solely as an environmental aspiration, but as an economic and clinical imperative. Over recent decades, the global healthcare sector has simultaneously expanded its environmental footprint and costs, revealing a system that is increasingly resource-intensive and financially strained. The evidence is clear: higher healthcare expenditure is consistently associated with greater greenhouse gas emissions, energy consumption, resource use, and pollution (Fig. 1). These environmental impacts are well-established drivers of kidney disease progression, revealing a direct and unsustainable link between volume-driven care and environmental harm.

**Figure 1:**
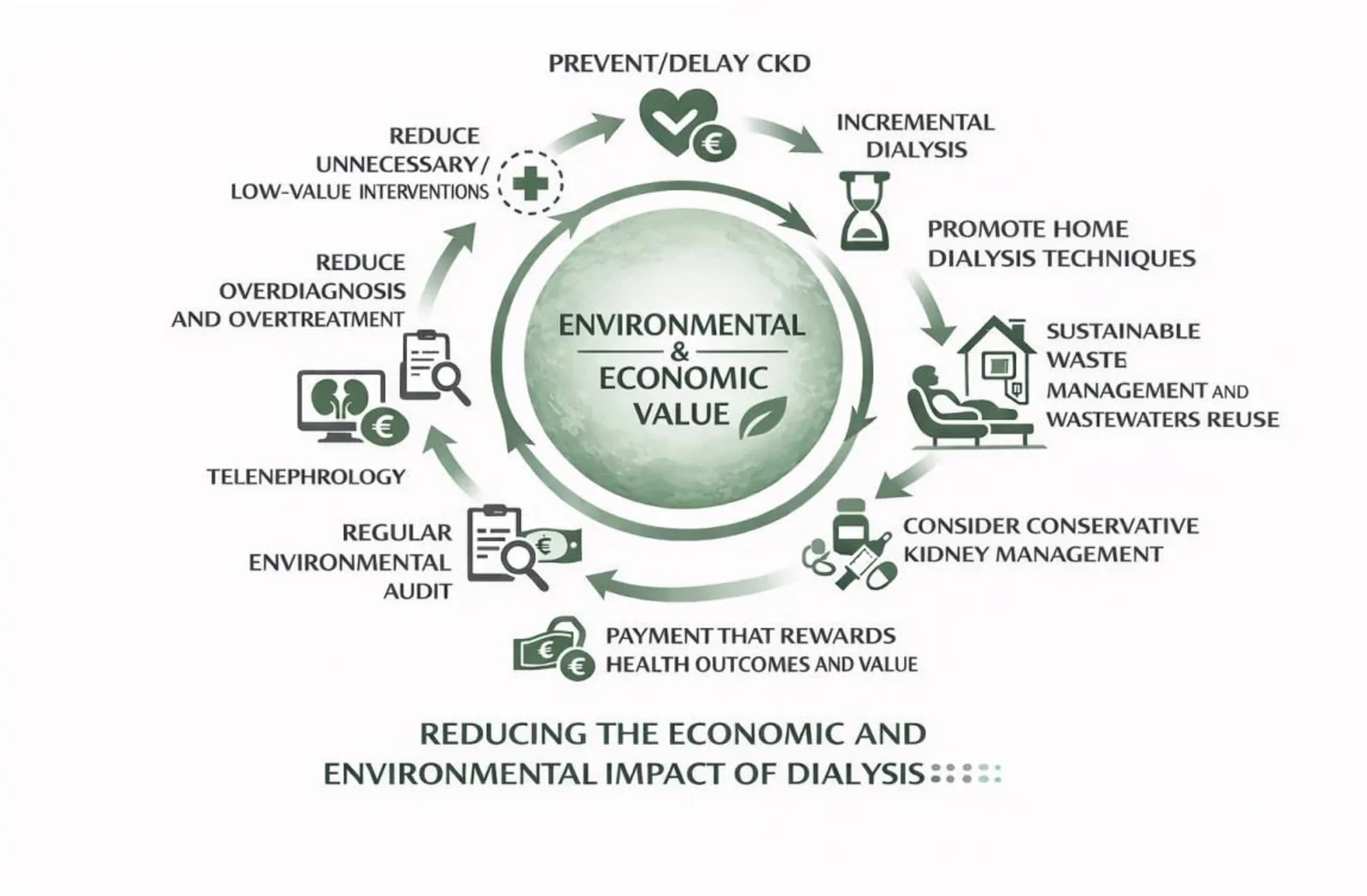
Reducing the economic and environmental impact of dialysis.

This imbalance is particularly evident in high-income countries, where healthcare-related carbon emissions are disproportionately high and cannot be explained solely by broader access or higher quality of care. A significant share of these emissions is now recognized to stem from unnecessary or low-value interventions. Dialysis illustrates this challenge, particularly when initiated in elderly patients with advanced comorbidities and frailty. Several studies have shown that, particularly in older and frail patients with advanced chronic kidney disease (CKD), conservative kidney management is associated with comparable or, in some cases, better patient-reported outcomes, including quality of life and symptom burden, as well as fewer hospitalizations, less intensive healthcare use, and a lower environmental impact than dialysis [1, 2]. Additionally, overdiagnosis and overtreatment of individuals with early and low-risk CKD may lead to repeated laboratory testing and clinical examinations. This occurs despite the low likelihood of progression to advanced and symptomatic stages of CKD, particularly in patients with slow disease progression, advanced age, and/or competing comorbidities that ultimately determine the overall prognosis and survival. For instance, in stable, nonprogressive stage 3 CKD, routine 3–6 month clinic visits may be safely replaced by risk-stratified follow-up based on the 5-year Kidney Failure Risk Equation.

Incremental dialysis offers a patient-centered alternative that may help preserve residual kidney function (studies have shown conflicting results), reduces costs, and lowers environmental impact compared with conventional thrice-weekly hemodialysis, while maintaining safety and quality of life in appropriately selected patients. Similarly, conservative kidney management represents a high-value option for selected patients, reducing hospitalizations, intensive interventions, and carbon emissions, while respecting patient preferences.

Dialysis, one of the most resource- and cost-intensive therapies in modern medicine, consumes vast amounts of water, energy, plastics, and pharmaceuticals. Yet, because of its scale, dialysis also represents one of the clearest opportunities for change. Even modest improvements in care delivery and resource management efficiency can result in substantial environmental gains while simultaneously reducing costs. In the setting of increasingly constrained healthcare budgets and escalating climate pressures, nephrology - and dialysis in particular - faces a critical inflection point, especially in low- and middle-income countries, where resource limitations are most prominent. Aligning environmental sustainability with cost-effective care through measures such as prevention-focused strategies, expansion of peritoneal dialysis, and implementation of lean dialysis practices (a set of management and operational methods focused on maximizing value while minimizing waste, including reduced dialysate flow rates, water reuse, and improved waste management) is both feasible and necessary; failure to do so risks perpetuating models of kidney care that are neither economically viable nor environmentally sustainable [3].

The importance of system-level efficiency is particularly evident in international comparisons. Healthcare costs tend to increase exponentially with the environmental footprint of health systems, a relationship illustrated by the contrast between China and Switzerland. While China has experienced a marked expansion of its healthcare resource footprint, this has not been matched by equivalent gains in access and quality of care. In contrast, Switzerland maintains one of the highest healthcare access and quality indices globally, with a comparatively stable resource footprint, reflecting greater efficiency and sustainability. Substantial cross-country differences in efficiency show that high-quality, accessible care can be delivered with markedly lower environmental footprints and optimized costs. For example, France, Japan, and the United States achieve similarly high healthcare access and quality scores, but with markedly different per-capita emissions (approximately 350, 1220, and 1720 kgCO_2_e, respectively), suggesting that gains in access and quality plateau beyond roughly 400 kgCO_2_e per capita and that further increases in emissions reflect inefficiencies rather than improved clinical outcomes (with the limitations of cross-country comparisons due to differences in health systems, energy resources, and calculation methods). This highlights the role of system design - particularly strong primary care, prevention, and coordinated service delivery - in achieving high performance with lower environmental impact [4, 5].

Beyond cross-country analyses, real-world experiences from individual health systems offer compelling evidence. In the UK, the Bradford Nephrology Department examined the cumulative impact of sustainability measures implemented over 17 years (interventions such as virtual clinics, online priming of hemodialysis machines, upgrade of water treatment systems, centralized dialysate acid delivery, use autoflow function, incremental and decremental dialysis practices, etc.), showing that interventions introduced since 2007 resulted in savings of >1000 tonnes of CO_2_e and roughly £2.8 million in reduced costs. This experience clearly illustrates that carbon reduction in healthcare can be closely aligned with cost efficiency, reinforcing the concept that environmental sustainability and economic stewardship are not competing objectives but mutually reinforcing goals within renal care [6].

At the patient level, prevention and slowing CKD progression represent a clear win-win, planet-friendly strategy and, beyond a moral imperative, a rational approach to the sustainability of any healthcare system, as preventing disease progression is inherently more efficient than treating its consequences. This is particularly relevant for patients at higher risk of progression or with rapidly declining kidney function, in whom the clinical and environmental benefits are greatest. As CKD progresses, resource requirements increase exponentially, with the transition to dialysis associated with >10-fold rise in carbon footprint (0.31 vs 3.9 tCO_2_e/year), a finding fully aligned with the European Renal Association ‘Protect Your Kidneys, Protect Your Future’ prevention campaign. This environmental impact is mirrored economically, as demonstrated by German cost analyses showing that, based on direct medical costs alone, dialysis care is ∼4.5 times more expensive than the management of CKD stage 4 [7].

A review of direct healthcare costs in 31 countries worldwide showed a similar magnitude of cost differences between CKD stage 5 and hemodialysis, with mean annual costs ranging from $3937 to $12 254 for nondialysis CKD stage 5 patients, compared with $26 096 to $79 678 per year for patients on hemodialysis. This cost difference is primarily driven by the high expenses associated with dialysis treatment and related hospitalization [8].

Within kidney care, incremental dialysis can be considered a cost-saving strategy through immediate reductions in treatment intensity, while prevention and risk-based CKD management are generally cost-effective due to delayed progression and improved outcomes; in contrast, infrastructure investments such as advanced water-treatment systems represent Capex (Capital Expenditure)- heavy cost-shifting interventions, with higher initial capital expenditure offset over time by reduced operational costs and environmental burden. Opportunities extend beyond clinical decision making to the management of dialysis-related waste streams. Dialysis waste management offers a clear opportunity to move from a linear to a more circular model of resource use, delivering both environmental and economic benefits to the society. Practical examples include the reuse of reverse osmosis reject water and the recovery of valuable nutrients from spent dialysate, which have been shown to reduce resource consumption while generating cost savings and new value streams (with a payback period of ∼12-42 months and no added risk to patients). Although these approaches vary in technical complexity, the available evidence suggests that some interventions are financially viable with relatively low investment and short payback periods. These examples illustrate the broader potential of circular economy strategies in dialysis care, among many other measures that can simultaneously reduce environmental impact and improve the economic efficiency.

Sustainability opportunities in nephrology also extend to rethinking the delivery of care. Telenephrology represents an alternative that remains underutilized and offers clear environmental advantages, including reduced patient travel and associated work absenteeism, while also generating economic savings for patients and their families, as demonstrated in cost analyses of telemedicine use in pediatric nephrology [9, 10].

To activate these opportunities, health systems must first quantify their impact. The sustainable management of dialysis units must be grounded in a fundamental principle: what cannot be measured cannot be managed. Embedding sustainability within performance management systems is therefore essential to support informed decision making, accountability, and continuous improvement. Organizations that adopt a proactive approach consistently demonstrate waste reduction, operational efficiency gains, lower resource-related costs, and improvements in institutional credibility and reputation. In contrast, the absence of standardized measurement frameworks leaves decision makers uncertain about which interventions to prioritize and limits their ability to assess the true effectiveness of implemented changes.

When evaluating technologies and operational processes in dialysis care, a life-cycle assessment is critical, extending beyond upfront capital costs to include maintenance requirements, consumable use, and broader system-level benefits, such as reduced staff workload and improved patient or workforce well-being. Addressing these complexities requires multidisciplinary collaboration, including expertize from outside the clinical domain, such as that of environmental engineers and waste management specialists. Within this framework, regular eco-reporting and clearly defined key performance indicators function as a vital link between management decisions and environmental outcomes, enabling structured environmental governance and the systematic implementation of operational sustainability initiatives [11, 12].

However, measurement alone is insufficient if broader economic incentives are misaligned. Conventional treatment-oriented, fee-for-service reimbursement models are based on the implicit assumption that a greater number of interventions equates to superior clinical outcomes. This paradigm creates a structural misalignment between economic incentives and sustainability. From a sustainability standpoint, escalating clinical activity is typically associated with increased consumption of materials, energy, and pharmaceuticals, with a corresponding increase in the environmental footprint, often in the context of marginal or diminishing gains in health outcomes. An important example of these trade-offs is the regional organization of dialysis services. While larger centralized dialysis centers may benefit from economies of scale and lower operational costs, they may also increase environmental impacts through longer patient travel distances and reduced accessibility. Conversely, a more decentralized network may reduce transport-related emissions and improve patient convenience but operate at higher unit costs. Identifying the optimal configuration therefore requires integrated decision making that considers not only financial costs, but also life-cycle environmental impacts, patient travel, equity of access, and clinical outcomes. Such multicriteria approaches can help planners identify context-specific solutions where economic efficiency and sustainability are best aligned.

A transition towards reimbursement frameworks that prioritize and reward health outcomes, value, prevention, and environmental sustainability - rather than the volume of services delivered - may represent a more sustainable approach to healthcare financing and create important incentives for greener dialysis practices. Mechanisms such as bundled payments for the entire dialysis episode, shared-savings models that reward prevention and the slowing of CKD progression, and outcomes-based contracts linked to clinical performance and resource efficiency could encourage providers to reduce waste. Nevertheless, these payment models must be carefully designed and monitored to avoid unintended consequences, including undertreatment, inappropriate cost cutting, or reduced access to dialysis services for vulnerable populations. While clinicians appropriately focus on optimizing outcomes for individual patients, sustainability should not be viewed as competing with patient-centered care. Safeguarding environmental health is intrinsically linked to protecting human health and has the potential to release resources that can be reinvested to enhance the quality of care, innovation, and core clinical priorities.

In conclusion, sustainable nephrology should be regarded not only as an environmental priority but also as an emerging economic strategy, a perspective that is still underrepresented in the literature. By integrating prevention, conservative kidney management, incremental dialysis, circular resource practices, and telenephrology, health systems may reduce emissions, water use, and waste generation while improving efficiency and revealing underappreciated opportunities for cost containment and improved resource allocation.

## Data Availability

Data sharing is not applicable to this article as no datasets were generated or analyzed during the current study.
